# Examining Constructs of Parental Reflective Motivation towards Reducing Unhealthy Food Provision to Young Children

**DOI:** 10.3390/nu11071507

**Published:** 2019-07-01

**Authors:** Brittany J. Johnson, Gilly A. Hendrie, Dorota Zarnowiecki, Elisabeth K. Huynh, Rebecca K. Golley

**Affiliations:** 1 Nutrition and Dietetics, College of Nursing and Health Sciences, Flinders University, Bedford Park 5042, South Australia, Australia; 2 School of Pharmacy and Medical Sciences, University of South Australia, Adelaide 5000, South Australia, Australia; 3 Health and Biosecurity Flagship, Commonwealth Scientific Industrial Research Organisation, Adelaide 5000, South Australia, Australia; 4 Early Prevention of Obesity in Childhood Centre for Research Excellence, Sydney 2006, New South Wales, Australia; 5 College of Health and Medicine, The Australian National University, Acton 2600, Australian Capital Territory, Australia

**Keywords:** unhealthy food, motivation, parents, early childhood, health action process approach model, self-efficacy, child nutrition

## Abstract

Parents are an ideal target to reduce children’s unhealthy food intake. Motivation is one component of behavior change; however, there is a paucity of research exploring parental motivation in unhealthy food provision. This study aimed to understand the relationships between, and relative importance of, constructs of parents’ reflective motivation and children’s intake of unhealthy foods. An online survey captured parent-rated reflective motivation constructs based on the health action process approach (HAPA) model, and children’s intake of unhealthy food using the short food survey. The HAPA model includes constructs of self-efficacy, risk perception, outcome expectancies, intention, and planning. Structural equation modelling was used to examine relationships between constructs and the HAPA model in its structural form. Four-hundred and ninety-five parents of three to seven-year olds completed the study. Model fit statistics (X^2^ = 210.03, *df* = 83, *p* < 0.001; Comparative fit index (CFI) = 0.96; Tucker Lewis index (TLI) = 0.94) supported suitability of the HAPA model. The HAPA model explained 9.2% of the variance in children’s unhealthy food intake. Constructs of self-efficacy (action to maintenance β = 0.69; maintenance to recovery β = 0.70; maintenance to planning β = 0.82) were found to be the most important constructs for reducing children’s unhealthy food intake, followed by planning (to unhealthy food intake β = −0.32) and intention (to planning β = 0.21). This study provides an initial insight into parental motivation and identifies primary intervention targets to enhance parental motivation to reduce unhealthy food provision, and subsequently children’s unhealthy food intake.

## 1. Introduction

It is well known that globally children overconsume unhealthy foods, whilst they concurrently fail to meet recommendations for healthy food groups, such as vegetables [1]. Recent Australian data shows 37% of four to eight-year old’s total energy intake is derived from unhealthy foods [2], with cakes and biscuits, takeaway foods, and savory pastries being key contributors [3]. At the same time, less than 5% of Australian children in this age group are meeting vegetable recommendations [4]. This dietary imbalance contributes to poor diet quality and can lead to an increased risk of nutrient deficiencies and chronic conditions, as well as obesity when excess energy is consumed [5].

There are numerous factors reported to influence children’s intake. Examples of such influences include children’s preferences, food availability and budget, and parent’s knowledge, attitudes, and beliefs [6]. Parents are a key influence, making parental provision a pragmatic behavior to target to moderate children’s unhealthy food intake [7,8]. Influences on parent provision can be broadly categorized as aspects of capability (e.g., knowledge and skills), opportunity (e.g., physical resources and social supports), and motivation (e.g., emotion/impulse driven or reflective) [9]. Michie and colleagues [9] suggest that for any behavior to occur we need the right balance of capability, opportunity, and motivation. When it comes to changing a behavior, even if we have the capability and opportunity to perform the new behavior, if we do not have the motivation, behavior change will not occur. Hence, parents’ motivation is an important aspect in initiating behavior change to reduce unhealthy food provision to their children.

Motivation can be described as reflective motivation, including self-conscious planning and evaluations; or automatic motivation, including habitual processes, desires, and emotion responses [9]. There are several constructs including self-efficacy, beliefs, outcome expectancies, intentions, goals, action planning, identity and optimism that are associated with reflective motivation, referred to hereafter as motivational constructs [9]. Whilst planning can be classified by theoretical domains that relate to psychological capability (i.e., behavioral regulation) and reflective motivation (i.e., goals) [9], for this study, action planning is conceptualized as contributing to reflective motivation. Few studies relating to young children’s dietary intake have comprehensively examined parental motivational constructs [10] and have often been limited to reporting associations between motivational constructs and parental intention, rather than children’s intake [11,12], or exploring constructs such as self-efficacy alone [13,14,15]. Greater understanding of reflective motivation can improve the development of interventions to enhance parents’ reflective motivation to support behavior change.

The health action process approach (HAPA) model provides a theoretical framework to comprehensively understand constructs contributing to parents’ reflective motivation [16]. In brief, the model consists of two phases, the motivational phase that captures constructs leading to intention, and the volitional phase that includes post-intentional constructs of self-efficacy and planning leading to the behavior itself [16]. The motivational phase of the model is akin to the theory of planned behavior, commonly used in past nutrition and health research [11,12,17,18,19]. The theory of planned behavior has been criticized for its poor ability to predict behavior, creating what is referred to as the intention–behavior gap [16,17]. The HAPA model aims to bridge this gap by including the post-intentional constructs, however this requires further testing.

The current study aimed to use the HAPA model to improve our understanding of the relationships between, and relative importance of, motivational constructs and children’s intake of unhealthy foods. Specific questions in relation to this aim were: Can the HAPA model be applied to understand parents’ reflective motivation for reducing provision of unhealthy foods? Which constructs within the HAPA model are of most importance to reducing parental provision of unhealthy foods? Does the HAPA model help to bridge the intention–behaviour gap? Understanding important motivational constructs will assist in designing interventions to reduce children’s unhealthy food intake.

## 2. Materials and Methods

This study used a cross-sectional design to measure motivational constructs influencing parents’ provision of unhealthy foods to their young children. Prior to commencing the study, parents were presented with a participant information sheet and consented to take part through the online survey tool. Ethics approval was obtained from the University of South Australia human research ethics committee (number 0000033798). Ethics endorsement was received from CSIRO health and medical human research low risk ethics committee (number LR 2/2015). The study was retrospectively registered with Australian New Zealand Clinical Trials Registry (ACTRN12617000603314).

### 2.1. Settings and Participants

The study was conducted online and included families across all states and territories in Australia. Recruitment of participants took part in two waves, one in April to August 2015 and one in March to July 2017 (to obtain a larger sample for analyses). Participants were recruited via online advertisements (i.e., Facebook, parenting magazines/forums), paper flyers, and snowball recruitment strategies, whereby participants referred others in their social network. An incentive was offered during the first wave only, with a chance to win one of ten double movie passes.

Parents of children aged three to seven years, living in Australia, fluent in written English were eligible for the study. Parents were excluded if their child was not within the specified age range (<3.0 or ≥8.0 years) at the time of the study, or if their child had a medical condition requiring a special diet inconsistent with the Australian dietary guidelines (e.g., cystic fibrosis) [20].

### 2.2. Variables

Children’s mean daily servings of unhealthy foods was the primary outcome used as a proxy measure of parent provision of unhealthy foods. Children’s intake of unhealthy foods was deemed a suitable proxy measure due to the associations between home food availability and children’s food intake [21,22]. Food and drink items were considered unhealthy foods as per the Australian dietary guidelines, for example: cakes, sweet biscuits, crisps, processed meats, and sugar-sweetened drinks [20].

Predictor variables were motivational constructs within the HAPA model (motivational and volitional phases). Constructs included: risk perception, positive and negative outcome expectancies, action self-efficacy, intention, maintenance self-efficacy, action planning, coping planning, and recovery self-efficacy [16].

Additional variables were collected to understand the parent sample. Parent variables included: age, gender, number of children, marital status, employment, education, household income, socio-economic status, and weight status. Child variables included: age, gender, and weight status.

### 2.3. Measurement Tools

Children’s intake of unhealthy foods was measured by the unhealthy foods subset of the short food survey—a food frequency questionnaire [23]. The unhealthy food subset has been found to have appropriate relative validity (correlation coefficient 0.44, *p* < 0.01) and reliability (correlation coefficient 0.87, *p* < 0.01) [23]. The questionnaire has been described in detail elsewhere [23]; in brief, the short food survey items selected included 15 questions about healthy foods and 20 detailed items about unhealthy foods. For example, ‘How often does your child usually eat savory pastries?’ with the response options of ‘each day’, ‘each week’, ‘each month,’ or ‘doesn’t eat savory pastries’; followed by ‘How many times does your child usually eat savory pastries?’ with a free text field for parents to enter the quantity. The frequency of unhealthy foods was converted to overall mean daily servings of children’s unhealthy foods, where one serving was equivalent to 600 kJ.

Predictor variables were collected using the parental food attitude questionnaire [24]. The parental food attitude questionnaire contains 57 items that measure 14 motivational constructs of the HAPA framework (motivational and volitional phases), specifically for parental provision of unhealthy foods. Our previous work has found the parental food attitude questionnaire to have good face and content validity [24]. The initial validation tests supported appropriate construct validity with predominately high factor loadings (motivational phase 0.43–0.89; volitional phase 0.53–0.85) and high internal consistency (Cronbach’s alpha motivational phase 0.77–0.88; volitional phase 0.85–0.92) [24].

Socio-demographic items were adapted or replicated from previous nutrition surveys and the Australian government census [25,26,27]. Parent self-reported weight and height were used to calculate body mass index (BMI; kg/m^2^) and classify weight status as underweight, healthy weight, overweight, or obesity [28]. Parent-reported child weight and height were converted to BMI *z*-scores by the least mean squares method, which adjusts for age and gender, using an add-in to Microsoft Excel [29]. Children’s BMI *z*-scores were then classified into weight status categories as underweight, healthy weight, overweight, or obesity using the international obesity task force definitions [30,31].

Socio-economic status was classified using the socio-economic indexes for areas (SEIFA) of relative advantage and disadvantage by matching parent-reported postcodes to SEIFA scores [32,33].

### 2.4. Data Collection Procedure, Bias, and Sample Size

All data were parent reported through an online survey (SurveyMonkey^®^). The survey took approximately 30–40 min to complete; an estimate time was presented to parents prior to the survey. The order of the questionnaire was designed to reduce the influence of social desirability bias and to reduce over- and under-reporting, for example by first collecting the whole of diet food frequency items to mask the focus on unhealthy food consumption.

Sample size was calculated based on the requirements for structural equation modelling. As the chance of error increases with the complexity of the model being tested in structural equation modelling, sample size is commonly calculated by the N:q rule, where q is the number of variables in the model [34]. A N:q ratio of 15 to 20 participants per variable is an accepted sample size guide [34,35]. The estimated sample size required for this study was 285 to 380 parents (assuming 19 variables).

### 2.5. Data Analysis

Data were exported from the online survey to Microsoft Excel (2011; Microsoft Corporation) for data cleaning, and where relevant parental food attitude questionnaire items were reverse scored. Normality was checked, extreme outliers were censored (to one unit above the closest plausible response within two standard deviations of the mean), and missing data were examined, then inputted using the full information maximum likelihood method in IBM^®^ SPSS^®^ (Version 25; SPSS Inc., Chicago, IL, USA). As Little’s missing completely at random test was significant (*p* = 0.21), a sensitivity analysis was performed for the final model, excluding participants with any missing data (Supplementary Material File S1). The outcome variable of children’s mean servings of unhealthy foods was square-root transformed to correct for skewness, and to be appropriate for use in structural equation modelling.

Structural equation modelling was performed to determine the relative importance of the motivational constructs and to test the suitability of the theorized HAPA model to capture parents’ intention to reduce unhealthy food provision. Structural equation modelling involves two stages: (1) measurement stage, and (2) structural stage, which includes confirmatory structural equation modelling when testing a theorized model, and exploratory structural equation modelling when testing a new model.

In the first stage, confirmatory factor analysis was performed initially for one factor models to examine the suitability of individual parental food attitude questionnaire sub-items at measuring each reflective motivation latent constructs, by examining model fit. Models were informed by preliminary exploratory factor analysis grouping parental food attitude questionnaire sub-items into motivation constructs. Once each construct was finalized, combined measurement models were tested for the HAPA motivational and volitional phases to complete the measurement stage of structural equation modelling.

In the second stage, each latent construct and measured variable (action self-efficacy, intention, and unhealthy food servings) were incorporated to form a theorized model (i.e., confirmatory structural equation modelling). Composite constructs were created for each latent construct to provide the statistical power to test the complex model; weighted composites were created using rescaled factor weight scores as constructs were found to be congeneric (i.e., unequal factor loadings for sub-items) [36]. Prior to running the final model, second order confirmatory factor analysis models were run when high correlations were observed between constructs, which aligned with the HAPA model structure. For example, high correlations were seen between Risk perception 1 and 2, so therefore all risk perception items were tested as a higher order construct for risk perception. The final model (Supplementary Materials Figure S1) consisted of 16 latent constructs (unmeasured; shown as ellipses) and three measured variables (shown as rectangles), including transformed mean unhealthy food servings as the outcome variable. In addition, a new model similar to the theory of planned behavior was tested using exploratory structural equation modelling to test whether the volitional phase constructs help to reduce the intention–behavior gap.

Confirmatory factor analyses and structural equation modelling were performed in IBM^®^ SPSS^®^ Amos (Version 25; SPSS Inc., Wexford, PA, USA) using the following model of fit assessment: chi-square (X^2^), comparative fit index (CFI), Tucker Lewis index (TLI), root mean square error of approximation (RMSEA), and standardized root mean-square residual (SRMR). A non-significant (*p* > 0.05) smaller X^2^ value is desirable but rarely achieved in large samples and complex models, therefore improvements were an indicator of better fit [37]. Comparative fit index and TLI were considered acceptable if >0.95, indicating normed and relative fit, respectively [38]. A RMSEA <0.05 and corresponding PCLOSE of >0.05 indicated suitable fit, taking into account the error of approximation in the population [37,39]. Standardized root mean-square residual was considered acceptable if <0.6 [37,39]. Multiple model fit statistics were evaluated to determine fit to provide robust assessment of model fit and overcome limitations of individual tests [34]. Bootstrap procedure of 500 samples for bias-corrected confidence intervals was used to obtain significance of overall indirect effects from within the model; though it should be noted that these estimates may be inaccurate with smaller sample sizes [34].

## 3. Results

### 3.1. Sample Characteristics

Seven-hundred and sixty-six parents commenced the online survey, with a 67% completion rate, resulting in 495 parents completing the entire questionnaire (2015: *n* = 167; 2017: *n* = 328; Supplementary Materials Figure S2). Twenty-eight participants were excluded due to ineligible child age (below 3.0 years old: *n* = 17; above 7.9 years old: *n* = 4), respondent was not the study child’s parent (*n* = 1), or the child had a medically indicated special diet precluding the Australian dietary guidelines (*n* = 6).

Parent and child characteristics are presented in Table 1. Participants were predominately mothers (94.9%), with a mean age of 36.8 (SD = 5.3) years, the majority were partnered (90.3%), with high education levels (73.7% tertiary degree or above) and high annual household income (52.3% $104,000 or more). There was representation from each socio-economic group, yet the lowest SEIFA tertile was underrepresented. Children had a mean age of 5.3 (SD = 1.6) years, and approximately half were female. Children’s intake of unhealthy foods was a median of 2.7 (IQR = 2.7) servings per day.

### 3.2. Stage One: Measurement Stage of Structural Equation Modelling

#### Motivational Latent Constructs

Confirmatory factor analyses outputs from each one-factor model are presented in Table 2 and Table 3. The measurement model resulted in 13 latent constructs consisting of 40 items and two single measured items, hence 42 items from the initial parental food attitude questionnaire. The initial parental food attitude questionnaire contained 57 items, four were removed during exploratory factor analysis (due to low communalities and one item for affecting the internal reliability), and 11 sub-items were removed during confirmatory factor analysis (Supplementary Materials Table S1 and S2). One to two sub-items were removed from most constructs, with three sub-items removed from each negative outcome expectancies, maintenance self-efficacy, and action planning constructs.

### 3.3. Stage Two: Structural Stage of Structural Equation Modelling

#### 3.3.1. Confirming the Health Action Process Approach Structural Model

Higher order latent constructs were proposed and tested for risk perception, maintenance self-efficacy, and planning based on the theoretical framework and correlations between latent variables (Table 4). A risk perception second order construct was initially tested with the four risk perception latent constructs, yet findings supported the second order construct to include only risk perception 1 and 2, both measures of absolute risk perception.

All first and second order latent constructs and measured variables were combined to form one model replicating the HAPA model (Supplementary Materials Figure S1). Model fit statistics supported adequate model fit. Chi-square was elevated and significant (210.03, *df* = 83, *p* < 0.001), as expected, given the large sample and complex model. Tucker Lewis index was near to an ideal fit at 0.94 (≥0.95 target), and CFI was ideal at 0.96. Root mean square error of approximation was acceptable at 0.056 with a related p value (PCLOSE) of 0.153, and SRMR was appropriate at 0.06.

**Figure 1:**
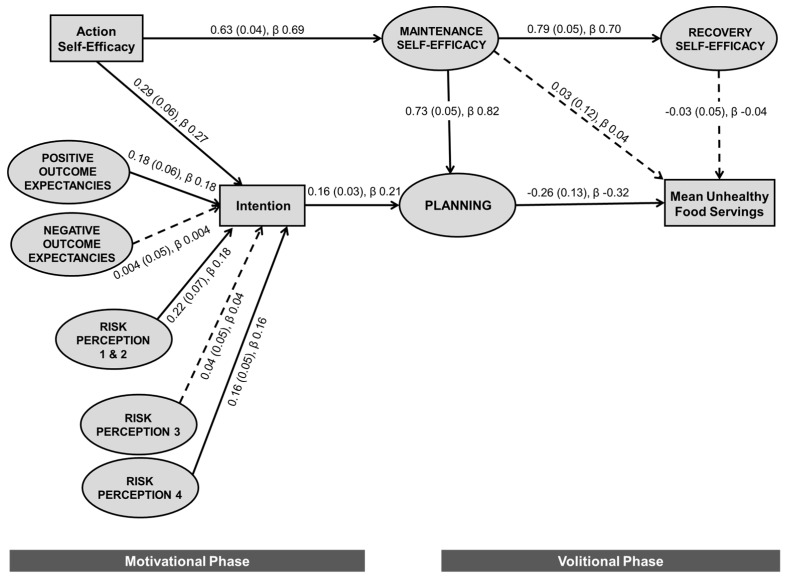
Final confirmatory structural equation modelling of the health action process approach model with unstandardized and standardized regression coefficients. Model fit: X^2^ = 210.033, *df* = 83, *p* < 0.001; CFI = 0.956; TLI = 0.936; RMSEA = 0.056, PCLOSE = 0.153; SRMR = 0.0601. Model explains 9.2% of the variance in mean unhealthy food servings. Weights presented as: unstandardized regression coefficient (standard error), standardized regression coefficient; rectangles represent measured constructs; ellipses represent latent constructs; solid line indicates statistically significant relationship (*p* < 0.05); dashed line indicates non-significant relationship.

Figure 1 presents the final confirmatory structural equation model. The six motivational phase constructs accounted for 32.8% of the variance in parental intention to limit children’s intake of unhealthy foods. The overall HAPA model was found to explain 9.2% of the variance in children’s mean servings of unhealthy foods (proxy measure for parental provision). The majority of paths within the model were significant, with the exception of negative outcome expectancies and risk perception 3—severity assessment to intention in the motivational phase, and maintenance self-efficacy and recovery self-efficacy to children’s servings of unhealthy foods in the volitional phase.

Maintenance self-efficacy to planning was the strongest relationship in the model, with a standardized regression coefficient (β) of 0.816 (unstandardized: *b* = 0.730, *p* < 0.001), suggesting that if you have higher maintenance self-efficacy (confidence to maintain limited provision in the face of barriers) you are more likely to also have higher levels of plans or strategies to manage both usual routine and more challenging circumstances (e.g., when visitors are present). There were also strong positive associations between parental confidence constructs. Amongst the motivational phase predictors, action self-efficacy was the strongest predictor of intention (β = 0.269, *b* = 0.289, *p* < 0.001). Intention had a positive association with planning, which in turn was inversely associated with unhealthy foods, implying that a higher level of planning was associated with lower servings of unhealthy foods.

The majority of overall indirect effects within the model were small, with the largest significant pathway being from action self-efficacy to recovery self-efficacy (β = 0.477, *b* = 0.496, *p* = 0.004), mediated by maintenance self-efficacy. Absolute risk perception 1 and 2 (β = 0.037, *b* = 0.035, *p* = 0.002), risk perception 4—for child (β = 0.032, *b* = 0.026, *p* = 0.002), and positive outcome expectancies (β = 0.037, *b* = 0.029, *p* = 0.002) all had significant indirect effects on planning, through intention. Whereas, negative outcome expectancies (β = 0.001, *b* = 0.001, *p* = 0.916) and risk perception 3—severity assessment (β = 0.007, *b* = 0.006, *p* = 0.390) did not. Action self-efficacy (β = 0.615, *b* = 0.505, *p* = 0.006) had a significant indirect effect on planning through intention or maintenance self-efficacy. Action self-efficacy was also indirectly inversely associated with children’s servings of unhealthy foods (β = −0.184, *b* = −0.124, *p* = 0.004) through intention or maintenance self-efficacy and planning. No other motivational phase construct had a significant indirect relationship with children’s servings of unhealthy foods.

#### 3.3.2. Exploring the Intention–Behavior Gap

**Figure 2:**
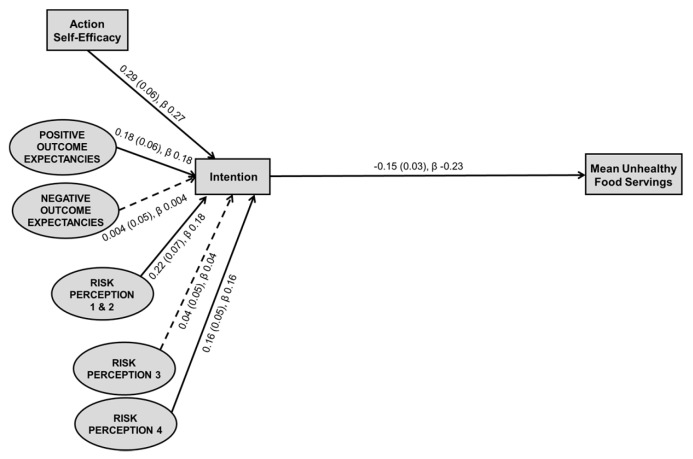
Exploratory structural equation modelling testing the predictive ability of intention to action unstandardized and standardized regression coefficients. Model fit: X^2^ = 55.294, *df* = 12, *p* < 0.001; TLI = 0.847; CFI = 0.949; RMSEA = 0.085, PCLOSE = 0.005; SRMR = 0.0496. Model explains 5.5% of the variance in mean unhealthy food servings. Weights presented as: unstandardized regression coefficient (standard error), standardized regression coefficient; rectangles represent measured constructs; ellipses represent latent constructs; solid line indicates statistically significant relationship (*p* < 0.05); dashed line indicates non-significant relationship.

A new model was tested using exploratory structural equation modelling to explore the intention–behavior gap. Figure 2 shows the model, composed of the HAPA motivational phase and children’s mean servings of unhealthy foods, hence removing the post-intentional volitional phase constructs of maintenance, recovery self-efficacy, and planning. The model fit statistics were appropriate (X^2^ = 55.294, *df* = 12, *p* < 0.001; TLI = 0.847; CFI = 0.949; RMSEA = 0.085, PCLOSE = 0.005; SRMR = 0.0496). A significant inverse direct association was observed between parental intention and children’s intake of unhealthy foods (β = −0.234, *b* = −0.147, *p* < 0.001). The new model accounted for 5.5% of the variance of unhealthy food servings. Inclusion of the volitional phase constructs (i.e., Figure 1) was seen to moderately bridge the intention–behavior gap (contributing 3.7% (of 9.2% total) of variance explained).

## 4. Discussion

The current study provides initial insight into motivational constructs contributing to parents’ unhealthy food provision using a theory-based approach. Specifically, we sought to understand the relationship between, and relative importance of, motivational constructs and children’s intake of unhealthy foods (proxy for provision). Findings supported the health action process approach (HAPA) model as a suitable framework to explain parents’ reflective motivation by capturing several motivational constructs. Parental action and maintenance self-efficacy (confidence to limit unhealthy food provision in ideal conditions, and to maintain confidence in the face of barriers, respectively) were found to play a key role in children’s unhealthy food intake, followed by planning and intention. Whereas, recovery self-efficacy (i.e., to again limit after unhealthy food provision) and negative outcome expectancies were not significant predictors of children’s unhealthy food intake, indicating that they may play less of a role in unhealthy food provision, or be areas where the model or measurement tool could be improved. Exploratory analyses highlighted additional benefits of the HAPA model, by the inclusion of post-intentional constructs, over the theory of planned behavior. Yet, the overall variance of children’s unhealthy food intake explained reinforced that there are other important aspects of parental provision or children’s intake of unhealthy foods not captured by motivation alone. Parents can be supported to reduce unhealthy food provision by designing interventions that enhance reflective motivation by targeting motivational constructs of self-efficacy, planning, and intention.

Model fit supported the HAPA model as an appropriate model to explain children’s unhealthy food intake by capturing motivational constructs contributing to parental reflective motivation. The model was found to account for nine percent of the variance in children’s unhealthy food intake. We had anticipated the explanatory ability of the model to be greater, but this may have been impacted by using child intake as a proxy measure for parents’ provision. Although there are no other studies using the HAPA model and children’s intake, the HAPA model has previously been found to account for 15–33% of the behavior of physical activity and healthy eating when investigating adult’s own behavior [40,41,42]. Several nutrition-related studies have explored some motivational constructs, such as attitudes, subjective norms, and perceived behavioral control, in relation to unhealthy foods or beverages, primarily using the theory of planned behavior; hence, somewhat comparable to the motivational phase of the HAPA model. Two studies have used the theory of planned behavior to understand sugar-sweetened beverage provision to preschoolers or adolescents, reporting the motivational constructs accounted for 48% and 32% of the variance in parental intention, respectively [11,43]; somewhat comparable to 33% of the variance in intention to reduce unhealthy foods in our study. Whereas, a meta-analysis of studies using the theory of planned behavior in various populations noted models accounted for, on average, 21% of the variance in intention to perform dietary behaviors [17]. Current findings support the use of the HAPA model to explain parents’ provision by capturing motivational constructs, yet the overall model could be improved to better explain children’s unhealthy food intake.

Parental confidence, or self-efficacy, was found to be the most important motivational factor. Specifically, our analyses found action self-efficacy (i.e., to limit unhealthy food provision in ideal conditions) and maintenance self-efficacy (i.e., to maintain limited provision in the face of barriers) the most important motivational constructs related to children’s unhealthy food intake. Studies in adults have also highlighted self-efficacy as the most important constructs with the HAPA model regarding physical activity and dietary change in intention and/or behavior [40,41,42,44]. Self-efficacy can enhance a parent’s mental strength and stamina to persist with performing or changing a behavior in line with their intentions. Previous studies in parents of young children and dietary intake have highlighted the importance of self-efficacy, noting inverse relationships between self-efficacy and children’s intake of select unhealthy foods and/or beverages [13,14,15]. In our study, recovery self-efficacy (i.e., to again limit after unhealthy food provision) was not associated with unhealthy foods intake in our sample. As self-rating of self-efficacy is highly influenced by past experiences [16], we speculate that, given intake data, parents in general have not yet reduced unhealthy foods to experience a set-back to enact recovery self-efficacy. Self-efficacy, specifically in ideal conditions (action self-efficacy) and in the face of barriers (maintenance self-efficacy) should form an initial intervention target.

Secondary intervention targets were identified to build comprehensive intervention supports, and to tailor intervention strategies and content, if parents already posed high levels of self-efficacy. Intention and planning (including action and coping planning) were found to be the next most important constructs within the HAPA model for reduced unhealthy food provision. Higher levels of intention were associated with higher levels of planning, which in turn was associated with lower child intake of unhealthy foods. Both intention and/or planning have also been identified as the second most important constructs in adults, specifically intention for physical activity, and intention and planning in dietary behaviors [40,41,44]. Intention alone may not be sufficient for a behavior to occur but including detailed plans such as implementation intentions and cue monitoring might help to reduce the intention–behavior gap [45]. Implementation intentions and cue monitoring involve problem solving and action planning to consider how and when intentions can be translated to behavior and identify prompts to encourage the behavior [45]. Intention and planning form complementary secondary intervention targets.

The intention–behavior gap was investigated in this study through exploratory structural equation modelling. We examined a modified version of the HAPA model, removing the volitional phase constructs, leaving a model representing the theory of planned behavior. These analyses revealed the addition of the volitional phase constructs within the HAPA model began to bridge this gap. Volitional constructs contributed nearly half of the total variance in children’s unhealthy food intake, explained by the model (i.e., 3.7% of variance). Albeit, the total variance explained by the HAPA model was small at 9.2%, signaling the need to capture other important aspects of children’s intake of unhealthy foods. Exploratory analyses provide support for advantages of the HAPA model, over the theory of planned behavior, to predict behavior.

### 4.1. Strengths and Limitations

The current findings need to be considered within the limitations of this study. Firstly, the cross-sectional design, in which the outcome of children’s unhealthy food intake was measured at the same time point as the predictor variables. Ideally, in future, children’s intake should be collected one month following parents’ stated intention as per the parental food attitude questionnaire phrasing. There are further limitations with parent-reported child intake, with a potential for social desirability bias and measurement error of the tool. Nonetheless, we did obtain a wide range in children’s servings of unhealthy foods, ranging from zero to 13 servings per day, with many exceeding dietary guideline recommendations. The non-random sampling approach also raises the potential for response bias. In addition, the resulting parent demographic of this sample was skewed towards mothers of higher education and income levels and did not adequately represent fathers’ motivation, hence limits generalizability of the findings to the Australian parent population [46]. Finally, there are other potentially important influences on children’s intake of unhealthy foods that were not captured within the HAPA model, such as parent feeding styles and practices, children’s preferences, and behavior. In addition, aspects of parents’ capability (i.e., knowledge and skills) and opportunity (i.e., physical resources and supports) were not measured given the scope of the current project, to focus on comprehensively examining motivation. Future studies can now use the prioritized important motivational constructs, in combination with capability and opportunity, to further explore unhealthy food behavior change.

There were, however, several strengths of this study. Firstly, the large sample recruited allowed for structural equation modelling of the complex model to be undertaken. Secondly, this study provided large quantitative data on motivation to be collected using a validated tool, though the reliability of the tool has not yet been assessed. Finally, the study was strengthened by the strong theoretical underpinning, allowing a comprehensive approach to understanding parents’ reflective motivation, and this provides direction for future intervention design.

### 4.2. Implications for Future Research and Practice

Several recommendations can be made for further research. Cross validation is required to confirm the suitability of the HAPA model, and it would be ideal for future work to recruit parent samples with a greater representation of parents of lower socio-economic background (under-represented in our sample). It would also be of interest to examine subgroups of parents, such as by intention status (e.g., pre-intenders, intenders, and actioners) or based on socio-demographic characteristics, to tailor supports to subsets of the parent population. Based on the current findings, future interventions can be designed to enhance parents’ motivation through prioritizing intervention strategies for self-efficacy, intention, and planning. Other theories, such as Bandura’s socio cognitive theory, could be used to design strategies to enhance parents’ self-efficacy [47]. In addition, tools such as the parental food attitude questionnaire can be used to evaluate changes in reflective motivation in such interventions. The refined parental food attitude questionnaire could also be used to assess a parent’s level of motivation in practice settings to tailor advice based on constructs obtaining low ratings; for example, towards enhancing self-efficacy, developing intention, or making plans to act on intention.

Lastly, from this exploration of motivation we are unable to determine what competing intentions parents may hold regarding food provision. For example, a recent review of qualitative studies synthesized parents’ motivations towards food provision into four key themes: promoting good health, building positive relationships, as well as practicalities and constraints, and emotional motivations [48]; the current study assumes parents are motivated by promoting good health, however these competing motivations require further exploration.

## 5. Conclusions

Parents’ reflective motivation to reduce unhealthy foods is needed to initiate a change in parents’ provision of unhealthy foods to their children. Current analyses provide guidance to prioritize the key constructs to be targeted in future interventions, namely action and maintenance self-efficacy to develop intention, and self-efficacy and planning for complex situations (i.e., in the face of barriers) to support parents to act on their intentions. Our results support the use of the HAPA model to explain motivational constructs contributing to parents’ reflective motivation towards reducing unhealthy foods. The overall model variance does, however, signal that there are other important factors that influence children’s unhealthy food intake not accounted for by motivation alone, such as parental capability or opportunity.

## Figures and Tables

**Table 1:** Descriptive characteristics of parents and children sampled (*n* = 495).

Characteristic	Parent	Characteristic	Child
Age, years (mean, SD)	36.8 (5.3)	Age, years (mean, SD)	5.3 (1.3)
Gender (%, count)		Gender (%, count)	
Male	5.1 (25)	Male	47.5 (235)
Female	94.9 (470)	Female	52.5 (260)
BMI ^1^ (mean, SD)	26.0 (5.5)	BMI z-score (mean, SD)	−0.15 (1.97)
Weight status (%, count)		Weight status (%, count)	
Underweight	1.3 (6)	Underweight	22.8 (107)
Healthy weight	52.7 (252)	Healthy weight	57.4 (270)
Overweight	28.0 (134)	Overweight	12.1 (57)
Obesity	18.0 (86)	Obesity	7.7 (36)
Number of children living at home (<18 years old) (%, count)		Education setting attendance (%, count)	
		Child care center	22.9 (122)
1	16.4 (81)	Family day care	1.9 (10)
2	54.9 (272)	Kindergarten	21.1 (112)
3	22.2 (110)	Primary school	50.4 (268)
4 or more	6.4 (32)	n/a ^5^	3.8 (20)
Relationship to child (%, count)		Median (IQR) mean servings of unhealthy foods	2.7 (2.7)
Mother	93.5 (463)		
Father	5.1 (25)		
Caregiver or other	1.4 (7)		
Marital status (%, count)		Residential area (%, count)	
Married/Living as married	90.3 (447)	Metropolitan	73.1 (362)
Single/Separated	9.7 (48)	Non-metropolitan	26.9 (133)
Education level (%, count)			
High school completion or below	7.6 (38)		
Tech or trade qualification	18.6 (92)		
Tertiary degree or higher	73.7 (365)		
Employment status (%, count)			
Employed	68.7 (340)		
Not in the workforce ^2^	31.3 (155)		
Annual household income ^3^ (%, count)			
Less than $52,000	14.4 (64)		
$52,000 to $103,999	33.4 (149)		
$104,000 and over	52.3 (233)		
SEIFA ^4^ Index of Advantage and Disadvantage (%, count)			
Low	19.2 (95)		
Medium	33.4 (165)		
High	47.4 (234)		

Abbreviations: BMI: body mass index; IQR: Interquartile range; SEIFA: Socio-Economic Indexes for Areas; SD: standard deviation. ^1^ Missing anthropometric responses for parent (*n* = 17) and child (*n* = 25); ^2^ Not in the workforce includes full time homemaker, student, volunteer work. ^3^ Missing income responses (*n* = 49) selected ‘I’d prefer not to answer’. ^4^ SEIFA scores were divided into tertiles: low (588–953), medium (954–1018), and high (1019–1191) as per Australian Bureau of statistics [32]. ^5^ n/a refers to children who do not attend an education setting.

**Table 2:** Final one factor model confirmatory factor analysis regression weights for motivational phase latent constructs.

Latent Constructs	Factor Loading ^1^	
Items	β	Unstandardized Coefficient (SE)
Risk perception 1—absolute risk		
child’s activity levels	0.793	0.779 (0.044)
child’s overall diet	0.878	0.952 (0.044)
Risk perception 2—absolute risk		
other children the same age	0.888	0.918 (0.039)
other children the same size	0.916	0.912 (0.037)
Risk perception 3—severity assessment		
being overweight	0.749	0.823 (0.045)
tooth decay	0.753	0.614 (0.033)
behavioral issues	0.789	0.709 (0.036)
too much energy and associated nutrients	0.794	0.733 (0.037)
Risk perception 4 ^2^—risk for child		
becoming overweight	0.927	0.879 (0.093)
developing tooth decay	0.687	0.556 (0.063)
Positive outcome expectancies		
be healthy	0.649	0.530 (0.038)
healthy eating habits	0.736	0.552 (0.034)
eat more fruit and vegetables	0.764	0.735 (0.044)
environmentally-friendly	0.380	0.331 (0.043)
Negative outcome expectancies		
throw a tantrum or pester	0.569	0.504 (0.044)
miss out on having treats	0.582	0.410 (0.035)
affect family time	0.564	0.404 (0.036)
overeat unhealthy foods when available	0.565	0.482 (0.043)
miss out on eating what their friends eat	0.602	0.465 (0.038)

Abbreviations: β: standardized regression coefficient; SE: standard error; ^1^ All regression weights were statistically significant with *p* < 0.001. ^2^ Note: Risk perception 4 model was run in combination with risk perception 3 to meet required degrees of freedom.

**Table 3:** Final one factor model confirmatory factor analysis regression weights for volitional phase latent constructs.

Latent Constructs	Factor Loading ^1^	
Items	β	Unstandardized Coefficient (SE)
Maintenance self-efficacy 1		
partner is undermining you	0.697	0.733 (0.043)
financial pressures	0.792	0.752 (0.037)
school/child care holidays	0.750	0.689 (0.037)
takes a long time to make it habit	0.749	0.666 (0.036)
food marketing on television	0.659	0.626 (0.040)
family time	0.609	0.562 (0.039)
Maintenance self-efficacy 2		
child is pestering for unhealthy foods	0.936	0.822 (0.031)
child is resistant to limiting unhealthy foods	0.949	0.819 (0.030)
Maintenance self-efficacy 3		
you are tired	0.944	0.902 (0.033)
having a very busy day	0.921	0.874 (0.033)
Action planning		
weekdays	0.914	0.805 (0.033)
weekend days	0.845	0.775 (0.035)
packing lunchbox	0.696	0.570 (0.034)
takeaway meals and snacks	0.612	0.587 (0.041)
Coping planning 1		
friends undermine my plans	0.924	0.877 (0.036)
relatives undermine my plans	0.813	0.738 (0.036)
Coping planning 2		
certain situations	0.768	0.689 (0.037)
set-backs when unhealthy foods have been provided	0.863	0.791 (0.037)
Recovery self-efficacy ^2^		
small relapse (2 days)	0.793	0.661 (0.032)
moderate relapse (2-6 weeks)	0.927	0.785 (0.030)
large relapse (weeks-months)	0.846	0.763 (0.034)

Abbreviations: β: standardized regression coefficient; SE: standard error; ^1^ All regression weights were statistically significant with *p* < 0.001; ^2^ Note: Recovery self-efficacy model was run in combination with maintenance self-efficacy constructs to meet required degrees of freedom.

**Table 4:** Second order confirmatory factor analysis regression weights for latent constructs of risk perception, maintenance self-efficacy, and planning ^1^.

Higher Order Construct	Factor Loading	
First Order Constructs	β	Unstandardized Coefficient (SE)
Risk perception		
Risk perception 1—absolute risk	0.894	0.893 (0.054)
Risk perception 2—absolute risk	0.820	0.819 (0.052)
Maintenance self-efficacy		
Maintenance self-efficacy 1	0.912	0.911 (0.041)
Maintenance self-efficacy 2	0.845	0.844 (0.040)
Maintenance self-efficacy 3	0.797	0.796 (0.041)
Planning		
Action planning	0.783	0.782 (0.046)
Coping planning 1	0.600	0.599 (0.050)
Coping planning 2	0.837	0.835 (0.048)

Abbreviations: β: standardized regression coefficient; CFI: comparative fit index; RMSEA: mean square error of approximation; SRMR: standardized root mean-square residual; SE: standard error; TLI: Tucker Lewis index; root. ^1^ All three final models were run in combination to meet required degrees of freedom. Model fit: X^2^ = 31.522, *df* = 16, *p* = 0.012; CFI = 0.991; TLI = 0.985; RMSEA = 0.044, PCLOSE = 0.629; SRMR = 0.0255.

## Supplementary Materials

The following are available online at https://www.mdpi.com/2072-6643/11/7/1507/s1, Figure S1: Theorized health action process approach model to examine using structural equation modelling; Figure S2: Flow of parent completion of the online survey; Table S1: Parental food attitude questionnaire motivational phase; Table S2: Parental food attitude questionnaire volitional phase; Supplementary File S1: Results from structural equation modelling sensitivity.

## References

[1] Popkin B.M., Adair L.S., Ng S.W. (2012). Global nutrition transition and the pandemic of obesity in developing countries. Nutr. Rev..

[2] Australian Bureau of Statistics Australian Health Survey: Nutrition First Results - Foods and Nutrients, 2011-12. http://www.abs.gov.au/ausstats/abs@.nsf/Lookup/4364.0.55.007main+features12011-12.

[3] Johnson B.J., Bell L.K., Zarnowiecki D., Rangan A.M., Golley R.K. (2017). Contribution of Discretionary Foods and Drinks to Australian Children’s Intake of Energy, Saturated Fat, Added Sugars and Salt. Children (Basel).

[4] Mihrshahi S., Myton R., Partridge S.R., Esdaile E., Hardy L.L., Gale J. (2019). Sustained low consumption of fruit and vegetables in Australian children: Findings from the Australian National Health Surveys. Health Promot. J. Austr..

[5] Guenther P.M., Casavale K.O., Reedy J., Kirkpatrick S.I., Hiza H.A., Kuczynski K.J., Kahle L.L., Krebs-Smith S.M. (2013). Update of the Healthy Eating Index: HEI-2010. J. Acad. Nutr. Diet..

[6] Johnson B.J., Hendrie G.A., Golley R. (2016). Reducing discretionary food and beverage intake in early childhood: A systematic review within an ecological framework. Public Health Nutr..

[7] Schrempft S., van Jaarsveld C.H., Fisher A., Wardle J. (2015). The Obesogenic Quality of the Home Environment: Associations with Diet, Physical Activity, TV Viewing, and BMI in Preschool Children. PLoS ONE.

[8] Yee A.Z., Lwin M.O., Ho S.S. (2017). The influence of parental practices on child promotive and preventive food consumption behaviors: A systematic review and meta-analysis. Int. J. Behav. Nutr. Phys. Act..

[9] Michie S., Atkins L., West R. (2014). The Behaviour Change Wheel: A Guide to Designing Interventions.

[10] Van Allen J., Kuhl E.S., Filigno S.S., Clifford L.M., Connor J.M., Stark L.J. (2014). Changes in parent motivation predicts changes in body mass index z-score (zBMI) and dietary intake among preschoolers enrolled in a family-based obesity intervention. J. Pediatr. Psychol..

[11] Tipton J.A. (2014). Using the Theory of Planned Behavior to understand caregivers’ intention to serve sugar-sweetened beverages to non-Hispanic Black preschoolers. J. Pediatr. Nurs..

[12] Andrews K.R., Silk K.S., Eneli I.U. (2010). Parents as health promoters: A theory of planned behavior perspective on the prevention of childhood obesity. J. Health Commun..

[13] Arsenault L.N., Xu K., Taveras E.M., Hacker K.A. (2014). Parents’ Obesity-Related Behavior and Confidence to Support Behavioral Change in Their Obese Child: Data From the STAR Study. Acad. Pediatr..

[14] Campbell K., Hesketh K., Silverii A., Abbott G. (2010). Maternal self-efficacy regarding children’s eating and sedentary behaviours in the early years: Associations with children’s food intake and sedentary behaviours. Int. J. Pediatr. Obes..

[15] Taveras E.M., Mitchell K., Gortmaker S.L. (2009). Parental confidence in making overweight-related behavior changes. Pediatrics.

[16] Schwarzer R. (2008). Modeling Health Behavior Change: How to Predict and Modify the Adoption and Maintenance of Health Behaviors. Appl. Psychol..

[17] McEachan R.R.C., Conner M., Taylor N.J., Lawton R.J. (2011). Prospective prediction of health-related behaviours with the Theory of Planned Behaviour: A meta-analysis. Health Psychol. Rev..

[18] Zoellner J.M., Porter K.J., Chen Y., Hedrick V.E., You W., Hickman M., Estabrooks P.A. (2017). Predicting sugar-sweetened behaviours with theory of planned behaviour constructs: Outcome and process results from the SIPsmartER behavioural intervention. Psychol. Health.

[19] Ajzen I. (1991). The Theory of Planned Behavior. Organ. Behav. Hum. Dec..

[20] National Health and Medical Research Council Australian Dietary Guidelines. https://www.nhmrc.gov.au/about-us/publications/australian-dietary-guidelines.

[21] Blaine R.E., Kachurak A., Davison K.K., Klabunde R., Fisher J.O. (2017). Food parenting and child snacking: A systematic review. Int. J. Behav. Nutr. Phys. Act..

[22] Campbell K., Abbott G., Spence A.C., Crawford D.A., McNaughton S.A., Ball K. (2013). Home food availability mediates associations between mothers’ nutrition knowledge and child diet. Appetite.

[23] Hendrie G.A., Viner Smith E., Golley R.K. (2014). The reliability and relative validity of a diet index score for 4-11-year-old children derived from a parent-reported short food survey. Public Health Nutr..

[24] Johnson B.J., Zarnowiecki D., Hendrie G.A., Golley R.K. (2018). Predictors of parental discretionary choice provision using the health action process approach framework: Development and validation of a self-reported questionnaire for parents of 4-7-year-olds. Nutr. Diet..

[25] Hendrie G.A., Cox D.N., Coveney J. (2008). Validation of the General Nutrition Knowledge Questionnaire in an Australian community sample. Nutr. Diet..

[26] Australian Bureau of Statistics How Australia Takes a Census. http://www.abs.gov.au/ausstats/abs@.nsf/mf/2903.0.

[27] Zarnowiecki D., Ball K., Parletta N., Dollman J. (2014). Describing socioeconomic gradients in children’s diets—does the socioeconomic indicator used matter?. Int. J. Behav. Nutr. Phys..

[28] World Health Organisation (2000). Obesity: Preventing and Managing the Global Epidemic.

[29] Pan H., Cole T.J. (2017). LMSgrowth, a Microsoft Excel Add-In to Access Growth References Based on the LMS Method. 2.2 ed.. https://www.healthforallchildren.co.uk/.2007.

[30] Cole T.J., Bellizzi M.C., Flegal K.M., Dietz W.H. (2000). Establishing a standard definition for child overweight and obesity worldwide: International survey. BMJ.

[31] Cole T.J., Flegal K.M., Nicholls D., Jackson A.A. (2007). Body mass index cut offs to define thinness in children and adolescents: International survey. BMJ.

[32] Australian Bureau of Statistics Socio-economic Indexes for Areas (SEIFA) 2011. http://www.abs.gov.au/AUSSTATS/abs@.nsf/Lookup/2033.0.55.001Main+Features12011?.

[33] Australian Bureau of Statistics Postcode 2012 to Remoteness Area 2011 in 1270.0.55.006—Australian Statistical Geography Standard (ASGS): Correspondences, July 2011. http://www.abs.gov.au/AUSSTATS/abs@.nsf/Lookup/1270.0.55.006Main+Features1July%202011?.

[34] Kline R.B. (2016). Principles and Practice of Structural Equation Modeling.

[35] Hendrie G.A., Coveney J., Cox D.N. (2011). Defining the complexity of childhood obesity and related behaviours within the family environment using structural equation modelling. Public Health Nutr..

[36] Khine M.S. (2013). Application of Structural Equation Modelling in Education Research and Practice.

[37] Schumacker R.E., Lomax R.G. (2004). A Beginner’s Guide to Structural Equation Modeling.

[38] Hu L., Bentler P.M. (1999). Cutoff criteria for fit indexes in covariance structure analysis: Conventional criteria versus new alternatives. Struct. Equ. Modeling.

[39] Byrne B.M. (2001). Structural Equation Modelling with AMOS: Basic concepts, applications, and programming.

[40] Barg C.J., Latimer A.E., Pomery E.A., Rivers S.E., Rench T.A., Prapavessis H., Salovey P. (2012). Examining predictors of physical activity among inactive middle-aged women: An application of the health action process approach. Psychol. Health.

[41] Parschau L., Barz M., Richert J., Knoll N., Lippke S., Schwarzer R. (2014). Physical activity among adults with obesity: Testing the Health Action Process Approach. Rehabil. Psychol..

[42] Renner B., Kwon S., Yang B.H., Paik K.C., Kim S.H., Roh S., Song J., Schwarzer R. (2008). Social-cognitive predictors of dietary behaviors in South Korean men and women. Int. J. Behav. Med..

[43] Riebl S.K., MacDougal C., Hill C., Estabrooks P.A., Dunsmore J.C., Savla J., Frisard M.I., Dietrich A.M., Davy B.M. (2016). Beverage choices of adolescents and their parents using the Theory of Planned Behavior: A mixed methods analysis. J. Acad. Nutr. Diet..

[44] Schwarzer R., Schuz B., Ziegelmann J.P., Lippke S. (2007). Adoption and Maintenance of Four Health Behaviors: Theory-Guided Longitudinal Studies on Dental Flossing, Seat Belt Use, Dietary Behavior, and Physical Activity. Ann. Behav. Med..

[45] Verhoeven A.A., Adriaanse M.A., de Vet E., Fennis B.M., de Ridder D.T. (2014). Identifying the ‘if’ for ‘if-then’ plans: Combining implementation intentions with cue-monitoring targeting unhealthy snacking behaviour. Psychol. Health.

[46] Australian Bureau of Statistics 2011 Census QuickStats. http://www.censusdata.abs.gov.au/census_services/getproduct/census/2011/quickstat/0.

[47] Bandura A. (1986). Social Foundations of Thought and Action: A Social Cognitive Theory.

[48] Rylatt L., Cartwright T. (2016). Parental feeding behaviour and motivations regarding pre-school age children: A thematic synthesis of qualitative studies. Appetite.

