# Supplementary material for: Examining Constructs of Parental Reflective Motivation towards Reducing Unhealthy Food Provision to Young Children
Source: Nutrients. 2019 Jul 1;11(7):1507. doi: 10.3390/nu11071507 (PMC6682954; doi:10.3390/nu11071507)
Supplement: Supplementary file 1 [file nutrients-11-01507-s001.pdf]

Examining constructs of parental reflective motivation towards reducing unhealthy food provision to young children

Brittany J Johnson [1, 2*], Gilly A Hendrie [3], Dorota Zarnowiecki [1,4], Elisabeth K Huynh [5], Rebecca K Golley [1,4,]

1 Nutrition & Dietetics, College of Nursing & Health Sciences, Flinders University, Bedford Park 5042, South Australia, Australia;
2 School of Pharmacy & Medical Sciences, University of South Australia, Adelaide 5000, South Australia, Australia
3 Health & Biosecurity Flagship, Commonwealth Scientific Industrial Research Organisation, Adelaide 5000, South Australia, Australia
4 Early Prevention of Obesity in Childhood Centre for Research Excellence, Sydney 2006, New South Wales, Australia
5 College of Health & Medicine, The Australian National University, Acton 2600, Australian Capital Territory, Australia
* Correspondence: brittany.johnson@flinders.edu.au

Supplementary Materials

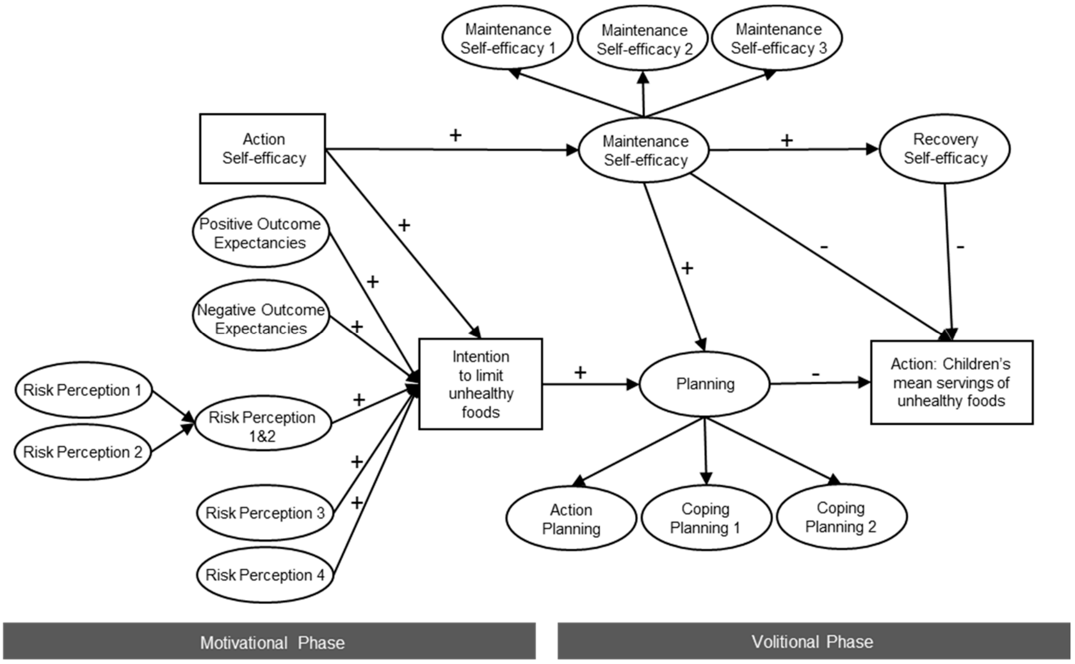

Figure S1. Theorized Health Action Process Approach model to examine using structural equation modelling. Adapted from Schwarzer [9]. Rectangles represent measured constructs; ellipses represent latent constructs; + represents hypothesized positive relationship and – represents a hypothesized negative relationship between variables.

Examining constructs of parental reflective motivation towards reducing unhealthy food provision to young children

Brittany J Johnson [1,2]*, Gilly A Hendrie [3], Dorota Zarnowiecki [1,4], Elisabeth K Huynh [5], Rebecca K Golley [1,4]

[1] Nutrition & Dietetics, College of Nursing & Health Sciences, Flinders University, Bedford Park 5042, South Australia, Australia;
[2] School of Pharmacy & Medical Sciences, University of South Australia, Adelaide 5000, South Australia, Australia
[3] Health & Biosecurity Flagship, Commonwealth Scientific Industrial Research Organisation, Adelaide 5000, South Australia, Australia
[4] Early Prevention of Obesity in Childhood Centre for Research Excellence, Sydney 2006, New South Wales, Australia
[5] College of Health & Medicine, The Australian National University, Acton 2600, Australian Capital Territory, Australia
* Correspondence: brittany.johnson@flinders.edu.au

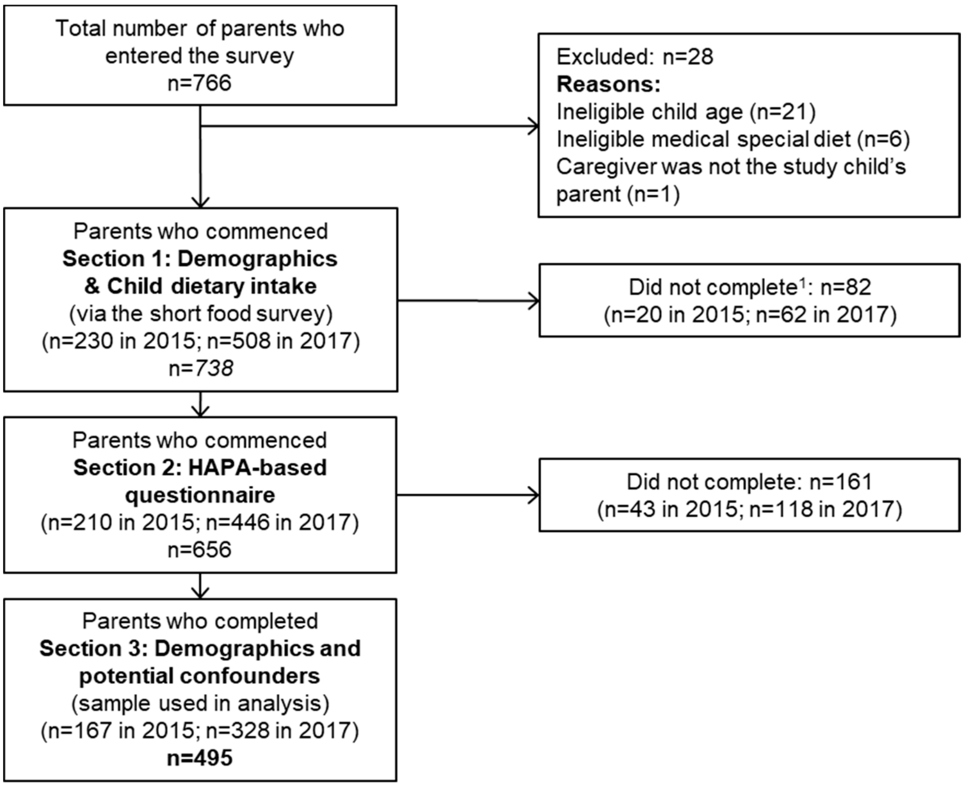

Figure S2. Flow of parent completion of the online survey

[1] Non-completers were excluded from all analyses.

Examining constructs of parental reflective motivation towards reducing unhealthy food provision to young children

Brittany J Johnson [1, 2*], Gilly A Hendrie [3], Dorota Zarnowiecki [1,4], Elisabeth K Huynh [5], Rebecca K Golley [1,4,]

[1] Nutrition & Dietetics, College of Nursing & Health Sciences, Flinders University, Bedford Park 5042, South Australia, Australia;
[2] School of Pharmacy & Medical Sciences, University of South Australia, Adelaide 5000, South Australia, Australia
[3] Health & Biosecurity Flagship, Commonwealth Scientific Industrial Research Organisation, Adelaide 5000, South Australia, Australia
[4] Early Prevention of Obesity in Childhood Centre for Research Excellence, Sydney 2006, New South Wales, Australia
[5] College of Health & Medicine, The Australian National University, Acton 2600, Australian Capital Territory, Australia
* Correspondence: brittany.johnson@flinders.edu.au

Table S1: Parental Food Attitude Questionnaire[1] motivational phase

Motivation construct	Final question	Scoring criteria	Scoring range
Risk perception 1 – absolute risk	1. Considering the extras you provide to your child from within the home, choose if you think this is lower to higher than the following statements.<br>*'Extras from within the home' refers to all home made or brought extras provided within the home, as well as those packed from the home e.g. lunchboxes and picnics.*<br>a) Compared with how active my child is the amount of extras I provide to my child is…<br>b) Compared with how healthy my child's overall diet is the amount of extras I provide to my child is…	5 = Lower<br>4 = Slightly lower<br>3 = Same<br>2 = Slightly higher<br>1 = Higher	2 - 10
Risk perception 2 – absolute risk	c) Compared with that of other children the same age as my child the amount of extras I provide to my child is…<br>d) Compared with that of other children the same size (weight and height) as my child the amount of extras I provide to my child is…		2 - 10
Risk perception 3 - severity assessment	2. For 4-7 year old children (i.e. similar age and size to your child), in general, how serious a concern do you think the following are:<br>a) being overweight<br>b) tooth decay<br>c) behavioural issues<br>d) too much energy (calories), saturated fat, added sugar and salt	1 = Not serious at all (can be ignored)<br>2 = Somewhat serious<br>3 = Moderately serious<br>4 = Serious<br>5 = Very serious (life threatening)	4 - 20
Risk perception 4 – risk for child	3. How much do you agree or disagree with the following statements. If I limit the extras I provide to my child, I think that I can reduce their chances of…<br>a) becoming overweight in the next 2-3 years<br>b) developing tooth decay	5 = Strongly agree<br>4 = Agree<br>3 = Neither agree nor disagree<br>2 = Disagree<br>1 = Strongly disagree	2 - 10

[1] The term 'extras' is used to describe unhealthy foods.

Examining constructs of parental reflective motivation towards reducing unhealthy food provision to young children

Brittany J Johnson [1, 2*], Gilly A Hendrie [3], Dorota Zarnowiecki [1,4], Elisabeth K Huynh [5], Rebecca K Golley [1,4,]

[1] Nutrition & Dietetics, College of Nursing & Health Sciences, Flinders University, Bedford Park 5042, South Australia, Australia;
[2] School of Pharmacy & Medical Sciences, University of South Australia, Adelaide 5000, South Australia, Australia
[3] Health & Biosecurity Flagship, Commonwealth Scientific Industrial Research Organisation, Adelaide 5000, South Australia, Australia
[4] Early Prevention of Obesity in Childhood Centre for Research Excellence, Sydney 2006, New South Wales, Australia
[5] College of Health & Medicine, The Australian National University, Acton 2600, Australian Capital Territory, Australia
* Correspondence: brittany.johnson@flinders.edu.au

Table S1: Parental Food Attitude Questionnaire motivational phase (continued)

Motivation construct	Final question	Scoring criteria	Scoring range
Positive outcome expectancies	4. How true are the following statements for you? If I limit my provision of extras from within the home…<br>a) …my child will be healthy (i.e. weight, teeth)<br>c) ...my child will continue healthy eating habits into adulthood<br>e) …my child will eat more fruit and vegetables<br>g) …I'll be seen to be environmentally-friendly	1 = Not at all true<br>2 = Somewhat true<br>3 = Mostly true<br>4 = Exactly true	4 - 16
Negative outcome expectancies	b) …my child will throw a tantrum or pester me for extras<br>d) …my child will miss out on having treats<br>f) …it will affect what we do in family time (i.e. movie nights, baking, celebrations etc)<br>h) …my child will overeat extras when they are available<br>i) ...my child will miss out on eating what their friends eat	4 = Not at all true<br>3 = Somewhat true<br>2 = Mostly true<br>1 = Exactly true	5 - 20
Action self-efficacy	5. How confident are you that you can limit the extras you provide to your child from within the home over the next month?<br>I am…	1 = Not at all confident<br>2 = Somewhat confident<br>3 = Moderately confident<br>4 = Extremely confident	1 - 4
Intentions	6. In the next month, I intend to start or continue to limit the extras I provide to my child from within the home.<br>I…	1 = Don't intend at all<br>2 = Somewhat intend<br>3 = Moderately intend<br>4 = Strongly intend	1 - 4

Examining constructs of parental reflective motivation towards reducing unhealthy food provision to young children

Brittany J Johnson [1, 2*], Gilly A Hendrie [3], Dorota Zarnowiecki [1,4], Elisabeth K Huynh [5], Rebecca K Golley [1,4,]

[1] Nutrition & Dietetics, College of Nursing & Health Sciences, Flinders University, Bedford Park 5042, South Australia, Australia;
[2] School of Pharmacy & Medical Sciences, University of South Australia, Adelaide 5000, South Australia, Australia
[3] Health & Biosecurity Flagship, Commonwealth Scientific Industrial Research Organisation, Adelaide 5000, South Australia, Australia
[4] Early Prevention of Obesity in Childhood Centre for Research Excellence, Sydney 2006, New South Wales, Australia
[5] College of Health & Medicine, The Australian National University, Acton 2600, Australian Capital Territory, Australia
* Correspondence: brittany.johnson@flinders.edu.au

Table S2: Parental Food Attitude Questionnaire volitional phase

Motivation construct	Final question	Scoring criteria	Scoring range
Maintenance self-efficacy 1	7. Some situations can make it hard to maintain certain behaviours. How confident are you, that you could limit providing extras to your child from within the home, even if…<br>e) …your partner is undermining you<br>f) …you have other financial pressures<br>g) …it is school/child care holidays<br>h) …it takes you a long time to make it habit<br>i) …your child sees food marketing on television<br>j) …you are having family time (i.e. movie night, baking, celebrations etc)	1 = Not at all confident<br>2 = Somewhat confident<br>3 = Moderately confident<br>4 = Extremely confident	6 - 24
Maintenance self-efficacy 2	a) …your child is requesting/ demanding/ fussing/ pestering you for extras<br>b) …your child is resistant to limiting extras		2 - 8
Maintenance self-efficacy 3	c) …you are tired<br>d) …you are having a very busy day		2 - 8
Action planning	8. Some parents would like to limit the extras they provide. How true are the following statements for you? I already have strategies for how to limit extras…<br>a) …at home on weekdays<br>b) …at home on weekend days<br>c) …when packing lunch for childcare / kindergarten / school<br>d) …when buying takeaway meals and snacks for eating at home	1 = Not at all true<br>2 = Somewhat true<br>3 = Mostly true<br>4 = Exactly true	4 - 16
Coping planning 1	9. Some parents would like to limit the extras they provide. How true are the following statements for you? I already have strategies for…<br>b) …how to manage when friends undermine my plans to limit extras<br>c) …how to manage when relatives (e.g. grandparents) undermine my plans to limit extras	1 = Not at all true<br>2 = Somewhat true<br>3 = Mostly true<br>4 = Exactly true	2 - 8
Coping planning 2	a) …how to deal with certain situations in order to stick to my intentions (e.g. where I know only extras may be available, when I'm in a hurry, school holidays)<br>d) …how to deal with set-backs when I provide extras outside of my intentions		2 - 8

Examining constructs of parental reflective motivation towards reducing unhealthy food provision to young children

Brittany J Johnson [1, 2*], Gilly A Hendrie [3], Dorota Zarnowiecki [1,4], Elisabeth K Huynh [5], Rebecca K Golley [1,4,]

[1] Nutrition & Dietetics, College of Nursing & Health Sciences, Flinders University, Bedford Park 5042, South Australia, Australia;
[2] School of Pharmacy & Medical Sciences, University of South Australia, Adelaide 5000, South Australia, Australia
[3] Health & Biosecurity Flagship, Commonwealth Scientific Industrial Research Organisation, Adelaide 5000, South Australia, Australia
[4] Early Prevention of Obesity in Childhood Centre for Research Excellence, Sydney 2006, New South Wales, Australia
[5] College of Health & Medicine, The Australian National University, Acton 2600, Australian Capital Territory, Australia
* Correspondence: brittany.johnson@flinders.edu.au

Table S2: Parental Food Attitude Questionnaire volitional phase (continued)

Motivation construct	Final question	Scoring criteria	Scoring range
Recovery self-efficacy	10. Sometimes we don't always stick to our intentions. Imagine you have increased the extras you provide to your child for some time. How confident are you about re-limiting the extras you provide to your child after…<br>a) …2 days (e.g. after a special occasion)<br>b) …2 to 6 weeks (e.g. after school holidays, Christmas period etc)<br>c) …weeks to months (e.g. after a period of change in family routine)	1 = Not at all confident<br>2 = Somewhat confident<br>3 = Moderately confident<br>4 = Extremely confident	3 - 12

Examining constructs of parental reflective motivation towards reducing unhealthy food provision to young children

Brittany J Johnson [1, 2*], Gilly A Hendrie [3], Dorota Zarnowiecki [1,4], Elisabeth K Huynh [5], Rebecca K Golley [1,4,]

[1] Nutrition & Dietetics, College of Nursing & Health Sciences, Flinders University, Bedford Park 5042, South Australia, Australia;

[2] School of Pharmacy & Medical Sciences, University of South Australia, Adelaide 5000, South Australia, Australia

[3] Health & Biosecurity Flagship, Commonwealth Scientific Industrial Research Organisation, Adelaide 5000, South Australia, Australia

[4] Early Prevention of Obesity in Childhood Centre for Research Excellence, Sydney 2006, New South Wales, Australia

[5] College of Health & Medicine, The Australian National University, Acton 2600, Australian Capital Territory, Australia

* Correspondence: brittany.johnson@flinders.edu.au

Supplementary File S1: Results from structural equation modelling sensitivity analysis

Sensitivity analysis of the HAPA-based structural equation model was performed on a sub-sample (n=339) excluding all respondents with any missing data (n=156). Model fit statistics (X^2=160.233, df=83, p<0.001; CFI=0.962; TLI=0.945; RMSEA=0.052, PCLOSE=0.355; SRMR=0.0606) supported appropriateness of the HAPA model. The model accounted for 11.0% of the variance in children's servings of unhealthy foods, with primarily similar regression coefficients. The largest paths were as per the primary analysis structural equation model, specifically *Maintenance self-efficacy* to *Planning* (β=.858, b=.723, p<0.001), and between constructs of self-efficacy (action self-efficacy to maintenance self-efficacy β=.719, b=.669, p<0.001; maintenance self-efficacy to recovery self-efficacy β=.728, b=.803, p<0.001).
